# Cohousing-mediated microbiota transfer from milk bioactive components-dosed mice ameliorate colitis by remodeling colonic mucus barrier and lamina propria macrophages

**DOI:** 10.1080/19490976.2021.1903826

**Published:** 2021-03-31

**Authors:** Cong Liu, Shimeng Huang, Zhenhua Wu, Tiantian Li, Na Li, Bing Zhang, Dandan Han, Shilan Wang, Jiangchao Zhao, Junjun Wang

**Affiliations:** a State Key Laboratory of Animal Nutrition, College of Animal Science and Technology, China Agricultural University, Beijing, China; b Academy of National Food and Strategic Reserves Administration, Beijing, China; c Key Laboratory of Animal Epidemiology of the Ministry of Agriculture and Rural Affairs, College of Veterinary Medicine, China Agricultural University, Beijing, China; d Department of Animal Science, Division of Agriculture, University of Arkansas, Fayetteville, AR, USA

**Keywords:** CMFG, colitis, intestinal homeostasis, gut microbiota, macrophages, cohousing

## Abstract

Human milk oligosaccharides (HMOs) and milk fat globule membrane (MFGM) are highly abundant in breast milk, and have been shown to exhibit potent immunomodulatory effects. Yet, their role in the gut microbiota modulation in relation to colitis remains understudied. Since the mixtures of fructo-oligosaccharides (FOS) and galacto-oligosaccharides (GOS) perfectly mimic the properties and functions of HMOs, the combination of MFGM, FOS, and GOS (CMFG) has therefore been developed and used in this study. Here, CMFG were pre-fed to mice for three weeks to investigate its preventive effect on dextran sodium sulfate (DSS) induced colitis. Moreover, CMFG-treated and vehicle-treated mice were cohoused to further elucidate the preventive role of the gut microbiota transfer in colitis. At the end of the study, 16S rDNA gene amplicon sequencing, short-chain fatty acids (SCFAs) profiling, transcriptome sequencing, histological analysis, immunofluorescence staining and flow cytometry analysis were conducted. Our results showed that CMFG pre-supplementation alleviated DSS-induced colitis as evidenced by decreased disease activity index (DAI) score, reduced body weight loss, increased colon length and mucin secretion, and ameliorated intestinal damage. Moreover, CMFG reduced macrophages in the colon, resulting in decreased levels of IL-1β, IL-6, IL-8, TNF-α, and MPO in the colon and circulation. Furthermore, CMFG altered the gut microbiota composition and promoted SCFAs production in DSS-induced colitis. Markedly, the cohousing study revealed that transfer of gut microbiota from CMFG-treated mice largely improved the DSS-induced colitis as evidenced by reduced intestinal damage and decreased macrophages infiltration in the colon. Moreover, transfer of the gut microbiota from CMFG-treated mice protected against DSS-induced gut microbiota dysbiosis and promotes SCFAs production, which showed to be associated with colitis amelioration. Collectively, these findings demonstrate the beneficial role of CMFG in the gastrointestinal diseases, and further provide evidence for the rational design of effective prophylactic functional diets in both animals and humans.

## Introduction

The intestine is a complex ecosystem harboring a dense and diverse microbial community called the gut microbiota, which co-evolved with the host to develop a mutualistic relationship.^1–1,2,3^ Loss of the equilibrium within this complex ecosystem has been shown to be implicated in numerous human diseases, such as inflammatory bowel disease (IBD) that affects up to six million individuals worldwide.^4–7^ IBD is a chronic inflammatory condition of the gastrointestinal tract, resulting from altered interactions between gut microbes and the intestinal immune system.^4,5^ There are two main IBD subtypes, Crohn’s disease (CD) and ulcerative colitis (UC), which localize in the small and large intestine, respectively, and are characterized by unique microbial composition.^8,9^ Patients with IBD exhibit major shifts in the gut microbial composition.^10^ Moreover, the composition of the gut microbiota directly or indirectly (via its metabolites) shapes the environment in the colon by modulating signaling, and immune response.^10–13^ The composition of the gut microbiota is affected by a wide range of factors, among which diet has been regarded as the most important modulators.^14^ Diet has been shown to participate in the regulation of the intestinal inflammation by modifying the gut microbiota composition and function, suggesting that dietary intervention can play a key role in alleviating IBD.^10,14–16^ However, novel and effective dietary intervention strategies are still lacking.

There are growing interest in the use of fecal microbiota for the treatment of patients with chronic gastrointestinal infections and IBD.^17,18^ Fecal microbiota transplantation (FMT) is gaining attention for the treatment of UC,^18–20^ since the donated gut microbial structure can repair the gut microbiota of the recipient and thus suppress harmful microbes overgrowth, promoting patient recovery.^19,21^ Growing studies demonstrated that cohousing promotes recovery from colitis via induction of epithelial cell proliferation and restoration of a functional epithelial barrier.^22,23^ In this study, we systematically addressed the role of diet in the successful microbiota transfer in murine models of colitis.

Human milk oligosaccharides (HMOs) and milk fat globule membrane (MFGM) are highly abundant in breast milk.^24,25^ HMOs resist gastrointestinal hydrolysis and digestion by pancreatic and brush-border enzymes, and are thus not absorbed in high amounts.^26^ Instead, they serve as prebiotic substrates for the gut microbes.^27^ Recent evidence has indicated that HMOs facilitate the gut microbiota establishment, promote intestinal development and stimulate immune maturation.^27–29^ Considering these beneficial effects and therapeutic potential of HMOs, mixtures of fructo-oligosaccharides (FOS), and galacto-oligosaccharides (GOS) have therefore been developed to resemble the molecular size distribution of the natural HMOs fraction found in human milk. Furthermore, they mimic the prebiotic from human milk, and are accessible to the gut microbiota.^30^ Therefore, the combination of FOS, and GOS could be used to examine the effects of HMOs on the gut microbial composition and intestinal epithelial barrier function.^31^ Also, MFGM has been shown to play an important role in modulating intestinal immune responses and the gut microbiota function.^32–35^ However, the roles of HMOs and MFGM in IBD remain unclear.

With respect to the underlying mechanism of the initiation and progression of IBD, past efforts have elucidated that the mucosal bacteria and other luminal antigens are associated with the immune responses induced by tissue-resident innate immune cells (e.g., dendritic cells and macrophages).^36,37^ Studies have shown that the gut microbial dysbiosis in IBD disturbs the innate immune system balance, as evidenced by altering the number and phenotype of dendritic cells and macrophages, thereby triggering a series of pro-inflammatory cascades in the colonic lamina propria.^38–40^ Markedly, the phenotype and functional state of macrophages are known to be closely associated with the intestinal environments.^41,42^ They are able to differentiate into two subtypes, including M1 and M2 with pro-inflammatory (e.g., IL-6, TNF-α, and IFN-γ) and anti-inflammatory (e.g., IL-10 and TGF-β) properties, respectively.^37,38,41^ Therefore, gut microbiota dysbiosis-induced macrophage polarization is a critical process in IBD development.^42^

In this study, we hypothesized that the combination of MFGM, FOS, and GOS (CMFG) regulates the colonic immune response, and especially reduce colonic infiltrating macrophages, via modulating gut microbial structure and function, leading to alleviated DSS-induced colitis. Given that gut microbiome has been implicated in the pathophysiology of IBD, and diet shapes the gut microbial structure, we investigated the impact of prophylactic CMFG intervention and microbiota transfer from the CMFG-treated mice to modulate the gut microbial composition, short-chain fatty acids (SCFAs) production, intestinal epithelial barrier function and colonic immune homeostasis in DSS-induced colitis in mice.

## Results

### Prophylactic CMFG intervention alleviated DSS-induced colitis

**Figure 1.**
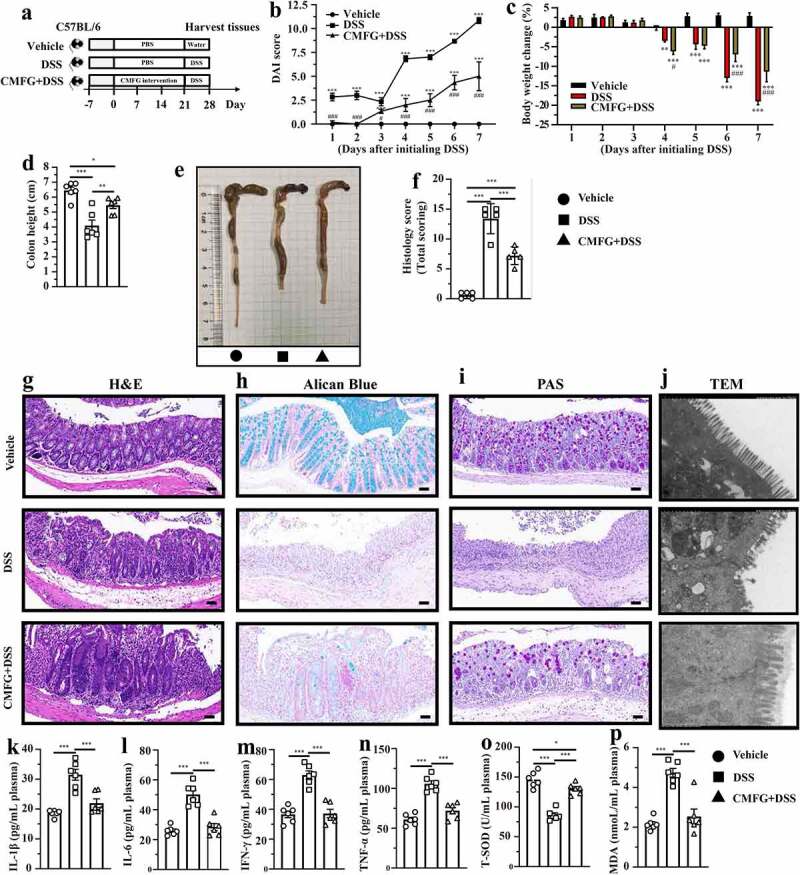
Prophylactic CMFG intervention prevents acute DSS colitis. A) experimental strategy, B) DAI score, C) body weight change, D) colon length, E) representative picture of the colon, F) summarized histological score, G) H&E staining of the colon, H) Alican blue staining, I) PAS staining, J) representative pictures of transmission electron microscopy. IL-1β (k), IL-6 (l), IFN-γ (m), TNF-α (n), T-SOD (o), MDA (p) level in the plasma. Asterisks denote significant differences (* *p* ≤ 0.05, ** *p* ≤ 0.01, *** *p* ≤ 0.001), *n* = 6 per group, data are represented as mean ± SEM

To investigate the effect of CMFG pre-supplementation on IBD, mice were administrated with either CMFG or vehicle (PBS) for 21 days followed by DSS treatment for 7 days (Figure 1a). As compared to the DSS group, mice in the CMFG + DSS group showed a lower disease activity index (DAI) score, lower weight loss, and colon length (Figure 1b,1,1d, and 1e). Moreover, CMFG increased crypts depth, reduced mononuclear cell infiltration and prevented mucosal damage in the colon tissue, resulting in a decreased histology score in DSS-treated mice when compared to mice in the DSS group (figure 1f,1g,1h, and 1i). Consistently, CMFG largely alleviated DSS-induced damage of brush borders and tight junctions in the colon (Figure 1j).

We next evaluated the effect of CMFG intervention on the inflammation and oxidative stress in IBD. To this end, the levels of inflammatory cytokines and oxidation products, and the activities of antioxidative enzymes in the colon and in the circulation were measured. As compared the DSS group, lower levels of IL-6, TNF-α, MPO, EPO and MDA and higher levels of T-AOC, CAT, T-SOD, and GSH-px were observed in the colon tissues of CMFG + DSS-treated mice (Figure S1). In line with these findings, CMFG + DSS-treated mice showed decreased levels of IL-1β, IL-6, IFN-γ, TNF-α and MDA, and increased levels of T-SOD in the circulation when compared to DSS-treated mice (Figure 1k-1p). Taken together, these results showed that CMFG treatment markedly ameliorated DSS-induced colitis.

### Prophylactic CMFG intervention modulate the colonic intestinal function in DSS-induced colitis

**Figure 2.**
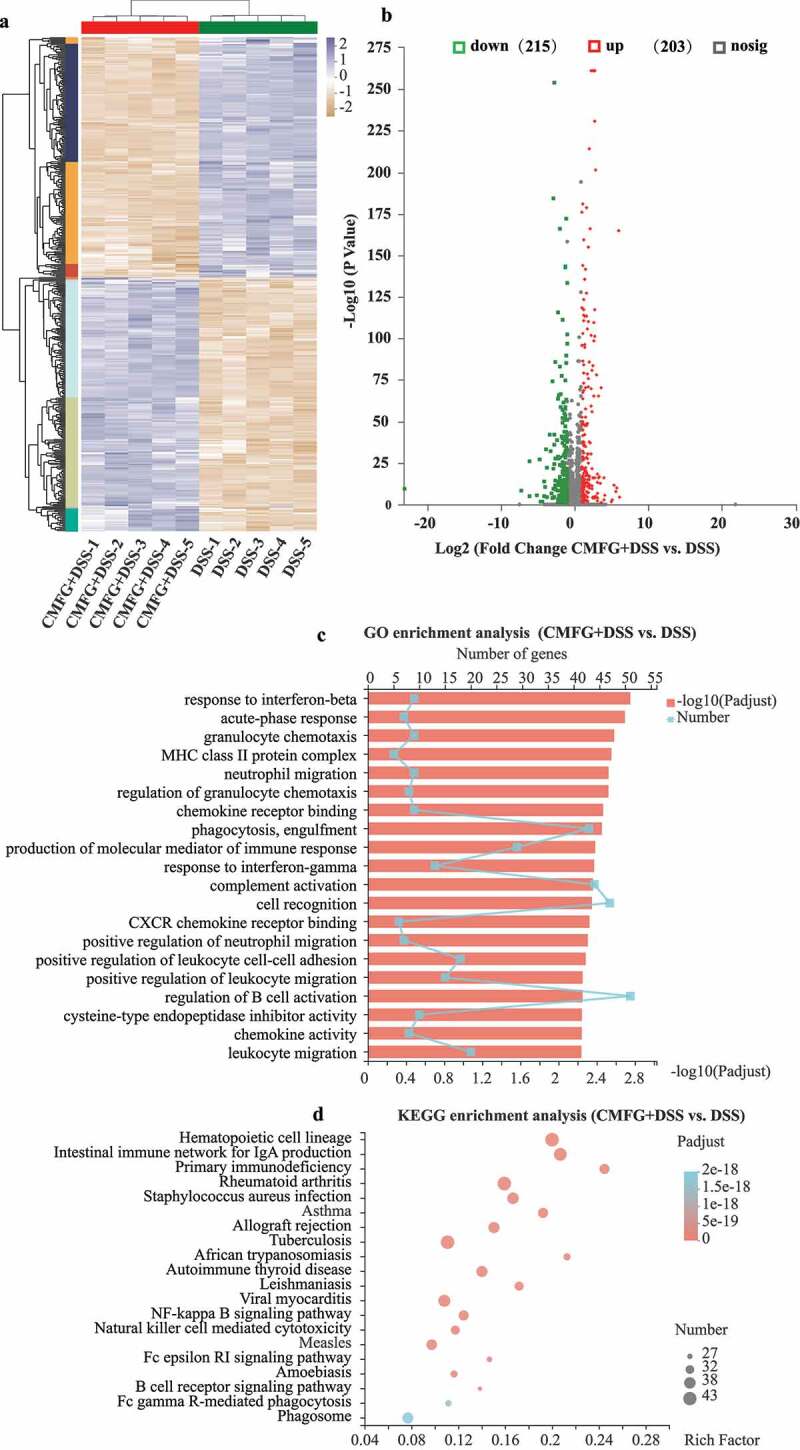
RNA-seq data exhibit distinct colonic function in colonic tissues. A) Heatmap summary of the differentially expressed genes. The scale bar shows the gene expression in each group. B) Volcano plot of differentially expressed transcripts with DSS and CMFG + DSS groups. C) GO enrichment of up-regulated and down-regulated genes in DSS vs. CMFG + DSS. D) KEGG enrichment of up-regulated and down-regulated genes in DSS vs. CMFG + DSS, *n* = 5 per group

To elucidate the underlying mechanisms of the CMFG induced improvement of DSS-induced colitis, RNA-seq were performed. A total of 215 genes were downregulated in the CMFG + DSS group, while 203 genes were upregulated when compared to the DSS group (Figure 2a and 2b). Gene Ontology (GO) enrichment analysis was subsequently performed to uncover the potential pathways of these differentially expression genes (DEGs). Interestingly, the most enriched pathways (CMFG + DSS vs. DSS) were closely related to immune responses, including the major histocompatibility complex (MHC) class II protein complex, neutrophil migration, response to interferon-gamma, and positive regulation of neutrophil migration. These findings suggest that CMFG mainly modulates the immune response in DSS-induced colitis (Figure 2c). The Kyoto Encyclopedia of Genes and Genomes (KEGG) pathway analysis further revealed that CMFG is involved in IgA production and NF-κB signaling pathway, which thus confirmed the functional role of CMFG in immunomodulation in IBD (Figure 2d).

### Prophylactic CMFG intervention alleviates DSS-induced colonic inflammation and intestinal barrier dysfunction

**Figure 3.**
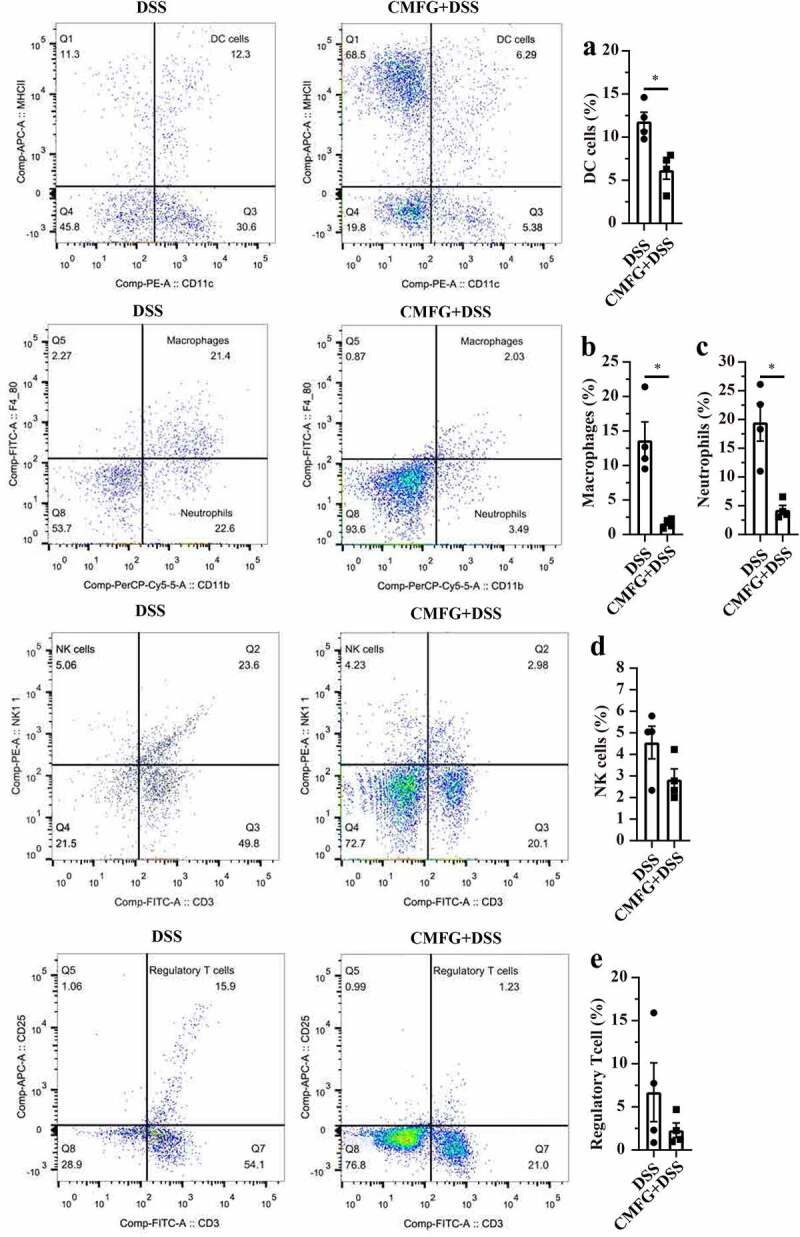
Prophylactic CMFG intervention altered the frequency of colon-infiltrating immune cells in colonic tissues. Representative plot of DC cells (a), Macrophages (b), Neutrophils (c), NK cells (d), and Treg cells (e) in the colonic tissues from DSS and CMFG + DSS groups. Asterisks denote significant differences (*p ≤ 0.05), *n* = 4 per group, data are represented as mean ± SEM

To understand how CMFG modulate colonic immunity, the profile of colonic immune cells was analyzed. As shown in Figure 3a,3b and 3c, the numbers of dendritic cells (DC), macrophages, and neutrophils were much lower in CMFG + DSS group than those in the DSS group.

**Figure 4.**
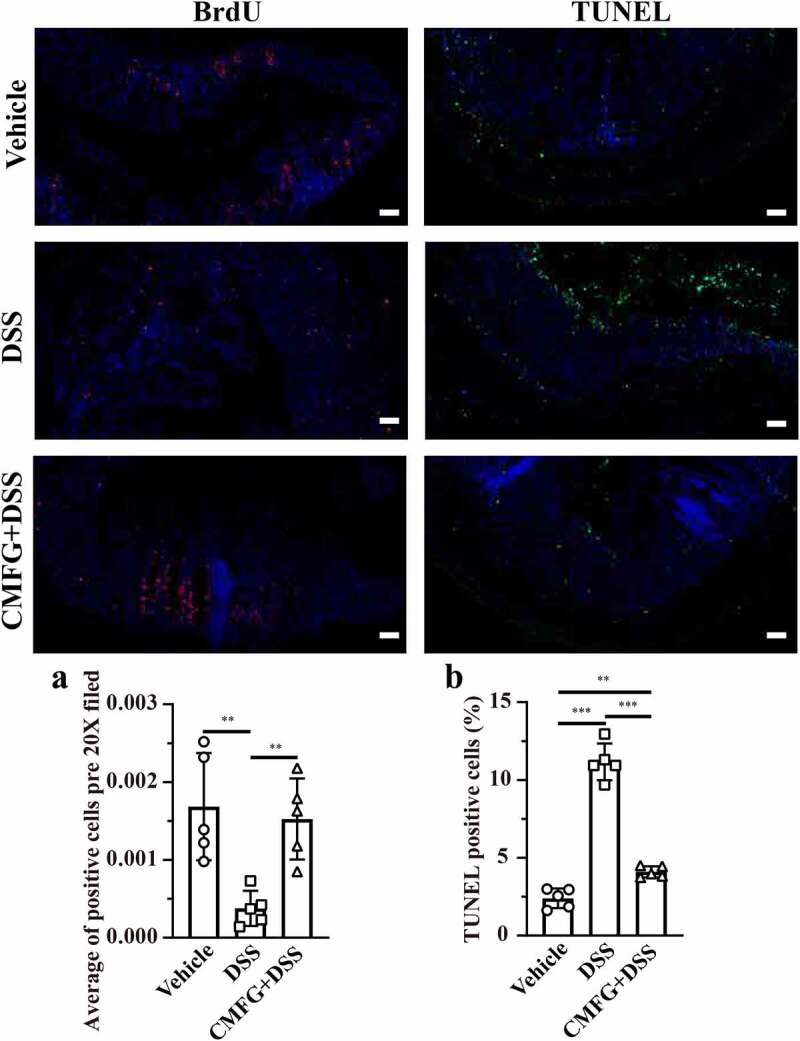
Prophylactic CMFG intervention alters the levels of cellular proliferation and apoptosis. Representative plot of cellular proliferation (a) and cellular apoptosis (b) in the colonic tissues among vehicle, DSS and CMFG + DSS groups. Asterisks denote significant differences (*p ≤ 0.05, ** *p* ≤ 0.01, *** *p* ≤ 0.001), *n* = 6 per group, data are represented as mean ± SEM

Since disturbed immune system balance has been shown to induce cell proliferation and apoptosis, we next investigated the impact of prophylactic CMFG intervention on the proliferation and apoptosis of colonic epithelial cells in DSS-induced colitis. Strikingly, colonic epithelial cells proliferation (represented by Bromodeoxyuridine, BrdU) was increased in CMFG + DSS-treated mice when compared to that in DSS-treated mice (Figure 4a). Moreover, CMFG largely inhibited DSS-induced epithelial cell apoptosis as demonstrated by reduced terminal deoxynucleotidyl transferase-mediated dUTP nick-end labeling (TUNEL) positive nuclei in colonic epithelium (Figure 4b). Furthermore, the effect of CMFG on the inflammatory status, oxidative stress and intestinal barrier in IBD was evaluated (Figure S2). Consistently, when compared with the vehicle group, DSS increased the colonic expression of genes involved in pro-inflammatory (*TNF-α, IFN-γ, IL-1β*, and *IL-6*), and antibacterial genes (*iNOS, NF-κB*, and *TLR-4*) processes, and decreased levels of *IL-10* (a key marker related to the anti-inflammation), and *ZO-1, Claudin-1*, and *Occludin* (key markers related to tight junctions), and *Mucin-1* and *Mucin-2* (key markers related to mucin secretion), while prophylactic CMFG intervention reversed these abnormal changes (Figure S2). These data indicated that the protective effect of prophylactic CMFG intervention was associated with reduced inflammatory responses, oxidative stress, and cell apoptosis in colonic tissues.

### Prophylactic CMFG intervention alters gut microbiota and its metabolites SCFAs production in DSS-induced colitis

**Figure 5.**
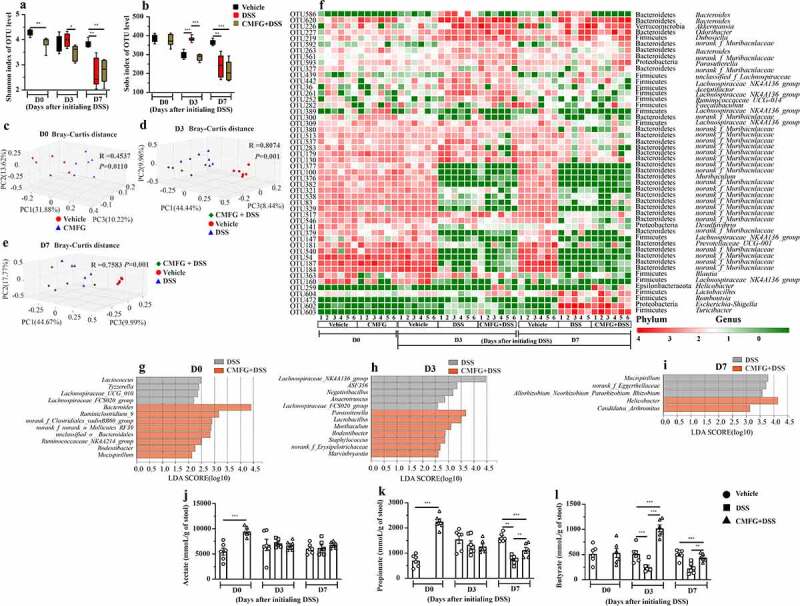
Prophylactic CMFG intervention alters the gut microbial composition and SCFAs production. A) Shannon index of OTU level. B) Sobs index of OTU level. C-E) PCoA plots assessed by Adonis analysis among these groups at day 0, at day 3, and at day 7, respectively. F) shows the relative abundance of microbial OTUs, classified at the phylum and genus level, in different groups. Linear discriminate analysis effect size (LEfSe) was performed to determine the difference in abundance at day 0 (g), day 3 (h), and at day 7 (i). SCFAs concentrations from feces among Vehicle, DSS, and CMFG + DSS groups are shown in J (acetate), K (propionate) and L (butyrate). Asterisks denote significant differences (*p ≤ 0.05, ** *p* ≤ 0.01, *** *p* ≤ 0.001), *n* = 6 per group, data are represented as mean ± SEM

Since the gut microbiota plays a pivotal role in the initiation and progression of the IBD, we next investigated the effect of CMFG on gut microbial composition in DSS-induced colitis. In the present study, we observed that the gut microbiota of CMFG-treated mice had a lower alpha diversity (Shannon index) than that of the vehicle-treated mice group at day 0 (Figure 5a). The gut microbial alpha diversity (Shannon index and Sobs index) differed between DSS-treated and CMFG + DSS-treated mice at day 3 (Figure 5a and 5b), while this difference disappeared at day 7 (Figure 5a and 5b). To extend our understanding of the role of gut microbial composition at the start of CMFG pre-supplementation (day 0), in the middle of DSS intervention (day 3), and further alterations occurred at the peak of inflammation (day 7), the principal coordinates analysis (PCoA) using Bray-Curtis metric distance was performed. The PCoA showed that the gut microbial structure of CMFG-treated mice differs from that of the vehicle-treated mice at day 0 and that of vehicle-treated and DSS-treated mice at day 3 and day 7 (Figure 5c-e and Figure S4).

Moreover, the gut microbial composition analysis showed that at the phylum level, all samples shared similar taxonomic communities and exhibited a relatively high abundance of the Firmicutes, Bacteroidetes, Proteobacteria, and Verrucomicrobia (figure 5f). At the genus level, the vehicle and CMFG groups shared a high abundance of the genera *Lachnospiraceae_NK4A136_group, Bacteroidetes*, and *Prevotellaceae_UCG-001* at day 0, while the CMFG + DSS-treated mice and DSS-treated displayed differential compositions at day 3 and 7 (figure 5f). Based on the linear discriminant analysis (LDA) of effect size (LEfSe) analysis, at day 0 (Figure 5g), *Bacteroides, Ruminiclostridium_9, Rodentibacter*, and *Mucispirillum* were more abundant in the gut microbiota of the CMFG-treated mice, while *Parasutterella, Lactobacillus*, and *Muribaculum* were enriched at day 3. At day 7, *Helicobacter* and *Candidatus_Arthromitus* were the most abundant microbes in the gut of the CMFG + DSS-treated mice (Figure 5h). However, *Mucispirillum* and *norank_f_Eggerthellaceae* (unidentified genus belonging to the family of *Eggerthellaceae*) were enriched in the DSS group at day 7 (Figure 5i). Considering the role of the gut microbiota in IBD, the differences of gut microbial composition between these two groups may be closely related to the phenotype changes of DSS colitis.

Next, the levels of SCFAs, key metabolites of the gut microbiota in the gut were measured. As compared to the vehicle group, CMFG-treated mice showed higher levels of acetate and propionate at day 0 (Figure 5j and 5k). Interestingly, CMFG treatment largely promoted the production of butyrate after 3 days of DSS intervention when compared to the DSS group (Figure 5l), while there is no difference in the levels of acetate and propionate (Figure 5j and 5k). At day 7, CMFG + DSS-treated mice showed increased propionate and butyrate levels when compared to DSS-treated mice (Figure 5k and 5l). Accordingly, CMFG pre-supplementation alters of the gut microbiota structure and promotes SCFAs production in DSS-induced colitis.

### Cohousing of CMFG-dosed mice promotes colitis symptoms recovery in DSS-induced mice

**Figure 6.**
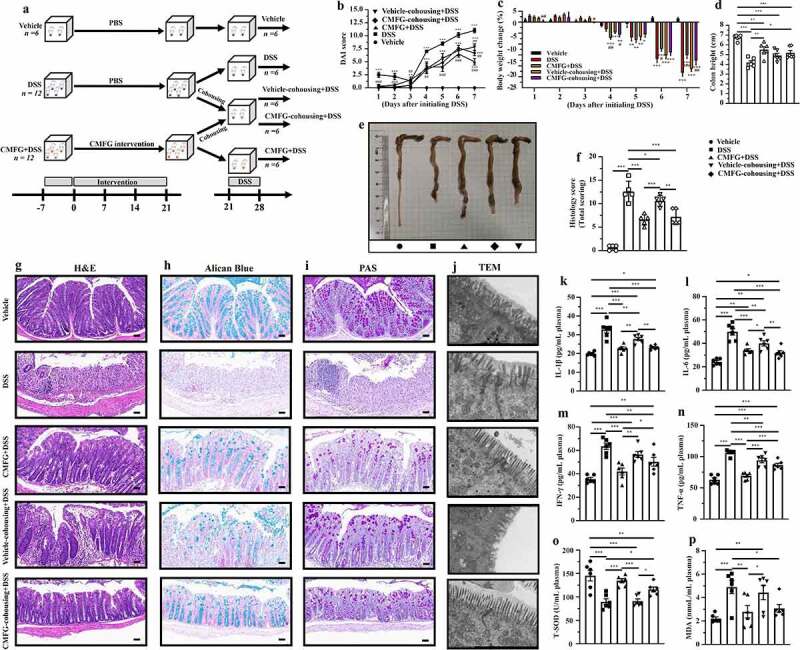
Cohousing of CMFG-dosed mice prevents acute DSS colitis. A) experimental strategy, B) DAI score, C) body weight change, D) colon length, E) representative picture of the colon, F) summarized histological score, G) H&E staining of the colon, H) Alican Blue staining, I) PAS staining, J) representative pictures of transmission electron microscopy. IL-1β (k), IL-6 (l), IFN-γ (m), TNF-α (n), T-SOD (o), MDA (p) level in the plasma. Asterisks denote significant differences (*p ≤ 0.05, ** *p* ≤ 0.01, *** *p* ≤ 0.001), *n* = 6 per group, data are represented as mean ± SEM

Although we observed that CMFG intervention alters the gut microbial composition and improves DSS-induced colitis, the association between CMFG-derived gut microbiota and DSS-induced colitis improvement was unclear. Therefore, an additional mice experiment was performed, in which mice treated with or without CMFG were cohoused during the DSS intervention period (Figure 6a). As expected, the DAI score and body weight loss of DSS-treated mice were decreased, and were comparable to their cohoused CMFG-treated counterparts (Figure 6b and 6c). Also, DSS-treated mice had longer colon length when they were cohoused with CMFG treated mice, and the colon length was comparable between these two groups. Likewise, under the cohousing conditions, DSS-treated mice exhibited lower histological score, which was within a similar range of that in CMFG-treated mice (Figure 6d,6e, and 6f). As compared to the controls (Vehicle-cohousing + DSS and DSS groups), mice in the CMFG-cohousing + DSS group exhibited less pro-inflammatory cell infiltration, relatively intact colonic architecture, less mucosal damage, and lower histology score (Figure 6g,6h, and 6i). Furthermore, DSS-induced damages of the brush borders and tight junctions were largely alleviated when they were cohoused with CMFG-treated mice (Figure 6j).

To address whether cohousing with CMFG-dosed mice displays anti-inflammatory and anti-oxidative characteristics, the levels of inflammatory and oxidative markers were measured in the colon tissues and circulation. Compared with DSS-treated mice, Vehicle-cohousing + DSS mice had lower concentrations of IL-1β, IL-6, and TNF-α in the plasma samples (Figure 6k,6l, and 6n). Also, the amount of IL-1β, IL-6, IFN-γ, TNF-α, and MDA in the CMFG-cohousing + DSS group were lower than those in the DSS group, while a higher concentrations of colonic T-SOD was presented in the CMFG-cohousing + DSS group (Figure 6o). Of note, compared with DSS-treated mice, Vehicle-cohousing + DSS mice had lower concentrations of inflammatory biomarker MPO in the colon (Figure S3C). Moreover, microbiota transfer from the CMFG-treated mice (Vehicle-cohousing + DSS mice) tended to reduce the levels of TNF-α, EPO, and MDA, and increase the concentrations of T-AOC, CAT, T-SOD, and GSH-px in DSS-treated mice (Figure S3). These results indicate that CMFG-derived gut microbiota transfer (via cohousing) alleviates DSS-induced colitis.

### Cohousing mediated transfer of the microbiota from CMFG-treated mice alleviates DSS-induced colonic inflammation through regulating macrophages and intestinal barrier function

**Figure 7.**
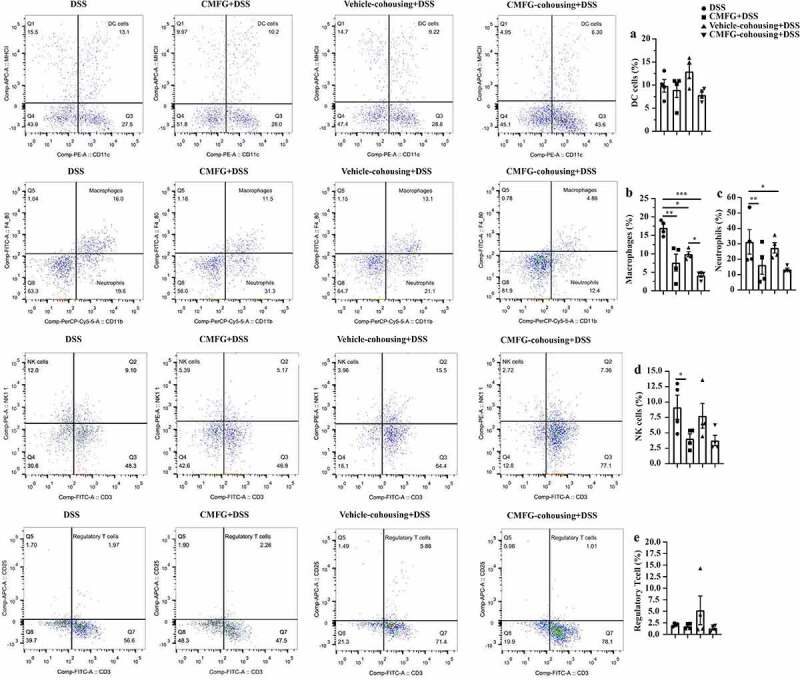
Cohousing of CMFG-dosed mice altered the frequency of colon-infiltrating immune cells in colonic tissues. Representative plot of DC cells (a), Macrophages (b), Neutrophils (c), NK cells (d), and Treg cells (e) in the colonic tissues among DSS, CMFG + DSS, vehicle-cohousing + DSS group and CMFG-cohousing + DSS. Asterisks denote significant differences (*p ≤ 0.05, ** *p* ≤ 0.01, *** *p* ≤ 0.001), *n* = 6 per group, data are represented as mean ± SEM

To further uncover the mechanisms of alleviated DSS-induced colitis mediated by the transfer of the gut microbiota from CMFG-dosed mice, FACS was performed. As compared to DSS-treated mice, microbiota transfer from the CMFG-treated mice (Vehicle-cohousing + DSS mice) showed significantly reduced macrophages and neutrophils in the colonic lamina propria, and the levels of these parameters are comparable to the CMFG + DSS group (Figure 7b and 7c).

**Figure 8.**
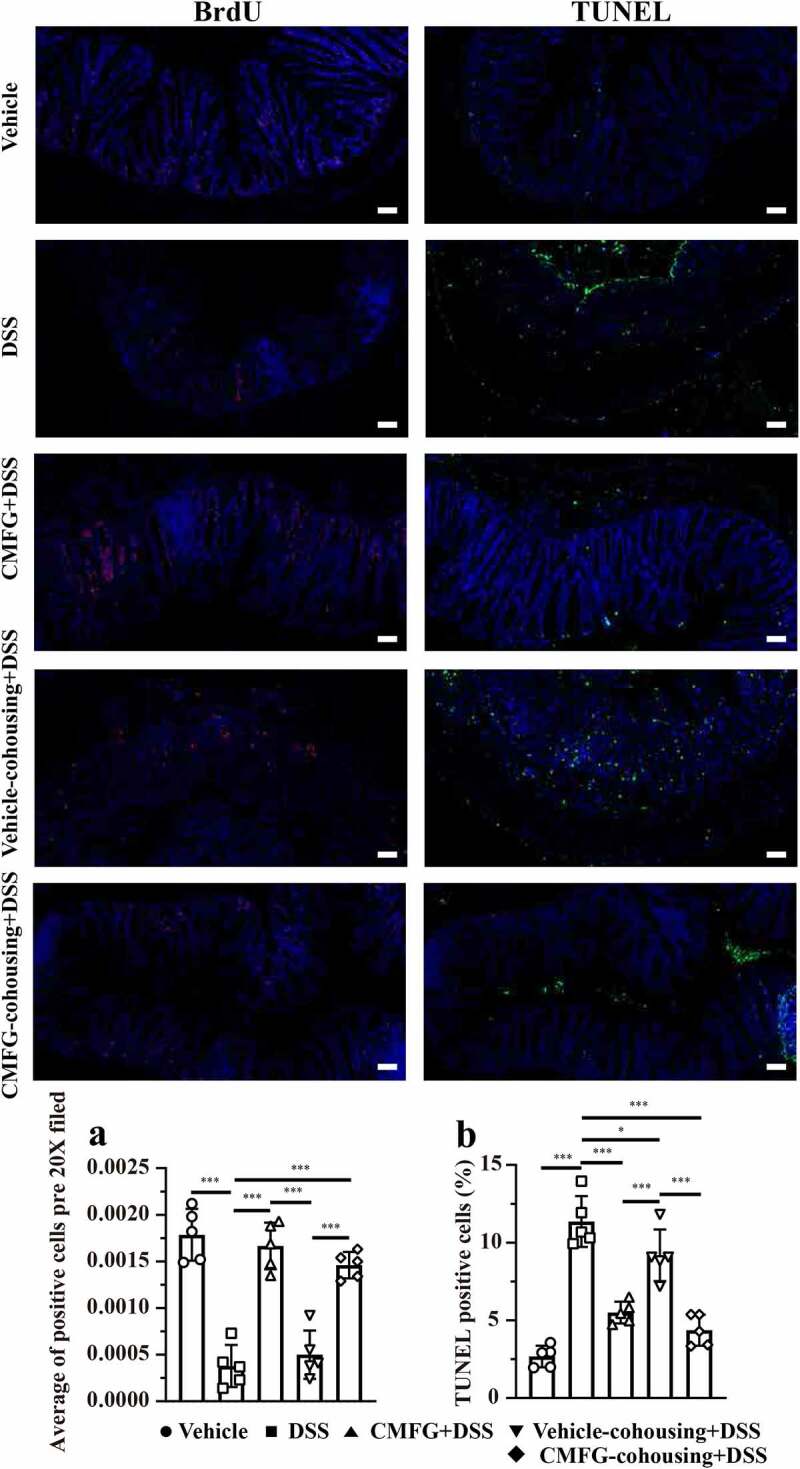
Cohousing of CMFG-dosed mice alters the levels of cellular proliferation and apoptosis. Representative plot of cellular proliferation (a) and cellular apoptosis (b) in the colonic tissues among DSS, CMFG + DSS, vehicle-cohousing + DSS group and CMFG-cohousing + DSS. Asterisks denote significant differences (*p ≤ 0.05, *** *p* ≤ 0.001), *n* = 5 per group, data are represented as mean ± SEM

**Figure 9.**
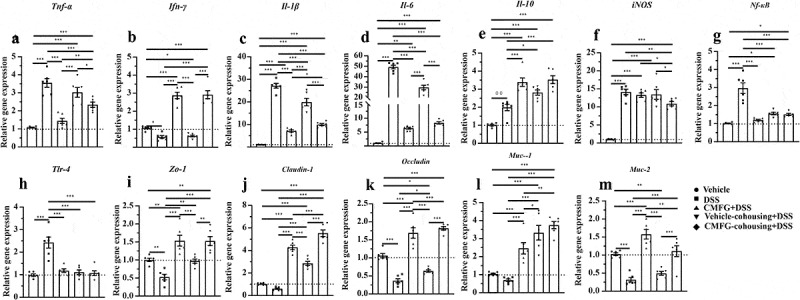
Cohousing of CMFG-dosed mice improved inflammation, oxidative, and barrier function genes expression in the colon. (a-e) The mRNA expression levels of the inflammation-related genes in the colon were analyzed by RT-qPCR. (f-h) The mRNA expression levels of the oxidative-related genes in the colon were analyzed. (i-m) The mRNA expression levels of the intestinal barrier function-related genes in the colon were analyzed. Asterisks denote significant differences (*p ≤ 0.05, ** *p* ≤ 0.01, *** *p* ≤ 0.001), *n* = 6 per group, data are represented as mean ± SEM

As shown in Figure 8a and 8b, the levels of BrdU positive cells and TUNEL positive cells were similar among CMFG + DSS and CMFG-cohousing + DSS mice groups. Compared with the DSS group, mice in the CMFG-cohousing + DSS group had higher concentrations of BrdU positive cells and lower level of TUNEL positive cells in the colon (Figure 8a and 8b). Strikingly, the TUNEL positive cells in colonic tissue were decreased in the Vehicle-cohousing + DSS group (Figure 8b), while Vehicle-cohousing + DSS mice tended to increase the levels of BrdU positive cells in DSS-treated mice (Figure 8a). Consistently, Vehicle-cohousing + DSS group showed lower levels of *IL-1β, IL-6, NF-κB* and *TLR-4*, as well as higher level of *IL-10* in the colonic tissues than those in the DSS group (Figure 9c,9d,9g,9h, and 9e). CMFG-cohousing + DSS group showed lower levels of *TNF-α, IFN-γ, IL-1β, IL-6, iNOS, NF-κB*, and *TLR-4*, as well as higher level of *IL-10* than those in the DSS group, and the levels of these parameters are comparable to the CMFG group (Figure 9a,9b,9c,9d,9f,9g,9h, and 9e). In addition, increased mRNA levels of *IL-10, ZO-1, Claudin-1, Occludin*, and *Mucin-1* in the colon tissues were observed in Vehicle-cohousing + DSS mice when compared to mice in the DSS group (Figure 9e,9i,9j, and 9k). Also, increased mRNA levels of *IL-10, ZO-1, Claudin-1*, and *Occludin*, as well as *Mucin-1* and *Mucin-2* in the colon tissues were observed in CMFG-cohousing + DSS mice when compared to the mice in the DSS group (Figure 9e,9i,9j,9k, and 9l). These data indicates that microbiota derived from CMFG-dosed mice has protective properties in maintaining intestinal barrier integrity.

### Cohousing mediated transfer of the microbiota from CMFG-treated mice alters gut microbiota composition and SCFAs production in DSS-induced colitis

**Figure 10.**
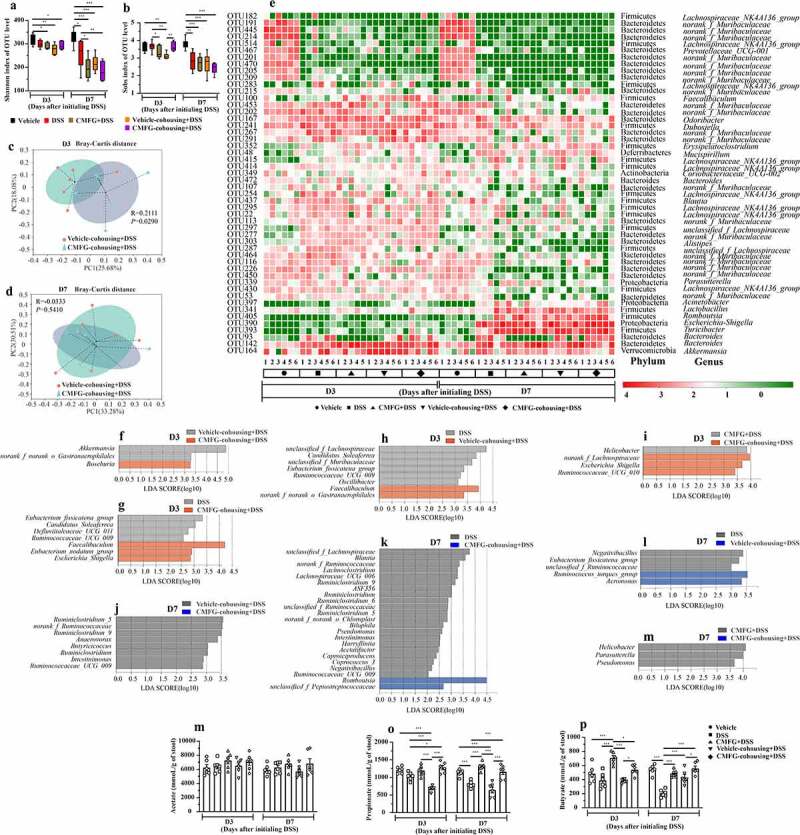
Cohousing of CMFG-dosed mice alters the gut microbial composition and SCFAs production. A) Shannon index of OTU level. B) Sobs index of OTU level. The PCoA of the gut microbiota was analyzed in different treatments at day 3 (c) and day 7 (d). E) shows the relative abundance of microbial OTUs, classified at the phylum and genus level, in different groups. Linear discriminate analysis effect size (LEfSe) was performed to determine the difference in abundance at day 3 (f-i), and at day 7 (j-m). SCFAs concentrations from feces in different groups are shown in N (acetate), O (propionate) and P (butyrate). Asterisks denote significant differences (*p ≤ 0.05, ** *p* ≤ 0.01, *** *p* ≤ 0.001), *n* = 6 per group, data are represented as mean ± SEM

To further confirm the functional role of CMFG-derived gut microbiota in alleviating IBD, the composition of gut microbiota and SCFAs levels were compared among DSS group, vehicle-cohousing + DSS group, and CMFG-cohousing + DSS groups. As shown in Figure 10C and 10d, different gut microbial structure and composition were observed between Vehicle-cohousing + DSS and CMFG-cohousing + DSS groups at day 3, while the gut microbial structure were similar between these two groups at day 7. As expected, cohousing treatment significantly altered the gut microbial structure and composition after 3 days of DSS intervention compared to the DSS group (Figure S5A). Interestingly, mice in the CMFG + DSS group, Vehicle-cohousing + DSS and CMFG-cohousing + DSS groups had similar gut microbial structure at day 3 and 7 (Figure S5C, S5D and S5F). Also, the main gut microbial composition in both phylum and genus levels were comparable between mice with vehicle-cohousing + DSS or CMFG-cohousing + DSS (Figure 10E). LEFSe analysis showed that *Roseburia* was enriched in CMFG-cohousing + DSS group, while *Akkermansia* was enriched in the Vehicle-cohousing + DSS group at day 3. As compared to the DSS group, the genera of *Faecalibaculum* and *Eubacterium_nodatum_group* were enriched in the CMFG-cohousing + DSS group at day 3. In addition, the genus of *Faecalibaculum* was enriched in the Vehicle-cohousing + DSS group when compared to the DSS group at day 3. Furthermore, compared to the CMFG-cohousing + DSS mice after 3 days of DSS intervention, the genera of *Ruminiclostridium, Anaerovorax, Butyricicoccus, Intestinimonas*, and *Ruminococcaceae_UCG_009* were significantly enriched in the Vehicle-cohousing + DSS mice (Figure 10J).

Next, we measured the concentrations of fecal SCFAs. As shown in Figure 10O, compared with the DSS group, the propionate levels were higher in the CMFG-cohousing + DSS group after 3 days and 7 days of DSS intervention, and the levels of this parameter were comparable to the CMFG + DSS group. Of note, the butyrate levels were higher in the Vehicle-cohousing + DSS and CMFG-cohousing + DSS groups after 7 days of DSS intervention (Figure 10P). These data thus suggest that microbiota from CMFG-treated mice could alter DSS-induced gut microbial structure and increase SCFAs production.

## Discussion

In this study, the effect of CMFG pre-supplementation on IBD was investigated in a DSS-induced colitis murine model. Our results demonstrated significant protective effect of prophylactic CMFG intervention on DSS-induced colitis, as evidenced by prevention of body weight loss and colon length shortening, reduced DAI score, decreased histology score, and improved mucosal barrier function and colonic ultrastructure. Of note, we also demonstrated that CMFG pre-supplementation alleviated colonic inflammation and suppressed the accumulation of colonic immune cells in the colonic lamina propria (e.g., macrophages) in a gut microbiota-dependent manner. Moreover, our data revealed that a broad and strong effect of DSS-induced of gut microbiota perturbation, and further elucidated that cohousing mediated transfer of the gut microbiota from CMFG-treated mice can alter DSS-induced abnormal changes and promote colitis recovery. Moreover, these data indicate that prophylactic CMFG intervention has a critical role in the prevention and treatment of IBD via the gut microbiota modulation.

Clinical studies have shown that patients with IBD had an aberrant gut microbial community,^43^ and the development of IBD is normally accompanied by abnormal gut microbiota alterations.^8,44^ In line with previous studies, we observed lower microbial alpha diversity in DSS-treated mice compared to the Vehicle group. Moreover, the gut microbial alpha diversity of mice in the CMFG and CMFG-cohousing groups differs from that in the DSS group. Furthermore, the PCoA analysis showed that the CMFG intervention prevented DSS-induced gut microbiota dysbiosis. Indeed, CMFG-treated mice, vehicle-cohousing mice, and CMFG-cohousing mice harbored microbial communities distinct from those in DSS-treated mice, indicating that CMFG cohousing treatment markedly modulates the gut microbiota structure. Consistently, LEfSe analysis showed different gut microbial composition in these groups. Intriguingly, in the middle of DSS intervention (day 3), the abundance of SCFAs-producing bacteria genus *Akkermansia* and *Lactobacillus* was enriched in the Vehicle-cohousing + DSS and CMFG + DSS groups, respectively. Recently, a large number of studies have shown that microbiota residing in the small and large intestine are adept at foraging mucins including those in host mucus.^45,46^ *Akkermansia* is effective at degrading mucin and often found at high abundance in the mucus layer.^47^ Previous results suggested that *Akkermansia* is associated with an anti-inflammatory role in gut health, which may be lost in IBD.^47^ Moreover, the presence of specific commensal bacteria such as *Lactobacilli* have been directly associated to the level of anti-inflammatory biomarker IL-10 and has been implicated in the maintenance of intestinal homeostasis.^48^ Of note, SCFAs-producing *Roseburia* was enriched in the CMFG-cohousing + DSS group after 3 days of DSS intervention. A link between *Roseburia* and gut health, including IBD, IBS and colon cancer, has been reported.^49,50^ Regarding inflammatory bowel disease, associations between *Roseburia spp*. and UC or CD have been described.^49,51^ *Roseburia*, the SCFAs-producing (mainly butyrate) bacteria, have been shown to exert anti-inflammatory properties, and play an important role in promoting intestinal motility and balancing intestinal epithelial immunity.^49,52^ As noted, *Roseburia* produces a significant amount of SCFAs (e.g., butyrate) from fermentable dietary carbohydrates.^49^ Together, our findings demonstrated that prophylactic CMFG intervention and microbiota transfer from the CMFG-dosed mice increased the abundance of SCFAs-producing commensal bacteria, which may aids to maintain the intestinal integrity and promotes the intestinal immune homeostasis.

Prebiotics intervention has been shown several advantages over probiotics, including increased resistance to pathogen infections and decreased risks.^53,54^ Previous studies showed that MFGM, FOS, and GOS promote the enrichment of microbes in the gut.^32,55,56^ Meanwhile, HMOs promote colonization and growth of the beneficial gut microbes, resulting in increased SCFAs production.^57–59^ Increasing evidence suggests that the microbial metabolites, such as SCFAs (e.g., butyrate), are important to maintain the intestinal barrier structure and function, which promotes mucosal homeostasis.^60–63^ For instance, butyrate, a histone deacetylase inhibitor, has been shown to maintain intestinal barrier function via downregulating claudin-2 in an IL-10-mediated manner.^64^ In addition, intestinal dysbiosis has been demonstrated to reduce butyrate in the intestinal lumen and fecal samples,^65^ causing a damaged intestinal barrier structure and stimulated initiate immune response.^66,67^ Of note, in experimental models of colitis, microbiota-derived SCFAs play an essential role in maintaining intestinal homeostasis due to their anti-inflammatory and anti-oxidative effects.^68^ In line with previous studies,^60,69^ we observed that the predominant microbes in the CMFG + DSS, Vehicle-cohousing + DSS and CMFG-cohousing + DSS groups were associated with SCFAs production and cohousing transfer of the gut microbiota from CMFG-treated mice reduced colonic inflammatory responses and increased SCFAs producing-bacteria.

Regarding to colonic immune cells, such as DC cells, macrophages, neutrophils, NK cells, and Treg cells, our data showed interesting patterns in response to CMFG pre-supplementation and CMFG-cohousing in the colitis settings. In this study, mice in the Vehicle-cohousing and CMFG-cohousing groups, showed reduced immune cells (e.g., macrophages) in the colonic lamina propria when compared with the DSS group. Growing studies showed that macrophages were abundant in colonic tissues, and exerted important functions in the immunity.^70^ Also, these results demonstrated that an increased apoptosis and lowered proliferation rate of intestinal epithelial cells are related to abnormal macrophages in murine colitis model.^70,71^ In line with our results, our data revealed that the TUNEL positive cells in colonic tissue were decreased in the Vehicle-cohousing + DSS group compared to the DSS group. Also, CMFG-cohousing + DSS mice had higher concentrations of BrdU positive cells and lower level of TUNEL positive cells than those in the DSS mice. These changes may be due to the decreased proliferation rate of intestinal epithelial cells induced by macrophage infiltrating.^70,71^ Macrophages infiltrating has been shown to produce tumor necrosis factor (TNF) and IL-6, thereby stimulating type 1 T helper (TH1) cell polarization. In the present study, the levels of TNF-α and IL-6 were decreased in the CMFG, Vehicle-cohousing and CMFG-cohousing groups. In addition, Treg cells have also been reported to promote the development of IBD.^72^ Studies have been showed that decreased Treg cells alleviates IBDs,^72^ which is consistent with our findings showing a trend in reducing Treg cells upon CMFG treatment. Furthermore, the cohousing experiment indicated that the gut microbiota alteration and macrophages reduction induced by CMFG-modulated microbiota transfer were closely related to the improved gut barrier function. Consistently, the reduced number of macrophages and the lowed expression of *TNF-α, IL-1β, IL-6, NF-κB*, and *TLR-4* were observed among CMFG, Vehicle-cohousing and CMFG-cohousing groups. Furthermore, comparable amounts of macrophages in CMFG +DSS and Vehicle-cohousing + DSS groups were observed, indicating that CMFG pre-supplementation or transfer of the microbiota from CMFG cohousing could improve the disturbed immune status in the colon of DSS-treated mice.

MFGM, FOS, and GOS have been shown to exhibit anti-inflammatory and anti-bacterial effects.^32,73–75^ Growing studies showed that inducible nitric oxide synthase (iNOS), a transcription factor, regulates the mRMA expression of anti-oxidative genes and produces a superoxide dismutase or a glutathione peroxidase in response to tissue oxidative stress followed by the accumulation of reactive oxygen species (ROS).^76,77^ In line with these findings, we observed decreased *iNOS, NF-κB* genes expression, and then reduced oxidative stress in the CMFG, Vehicle-cohousing and CMFG-cohousing groups. Furthermore, ROS-mediated oxidative stress and inflammation are known to reciprocally affect each other through intracellular signaling pathways, such as TLR4 and NF-κB.^78,79^ In our study, we demonstrated that the down-regulation of the *NF-κB* and *TLR-4* genes in the CMFG, Vehicle-cohousing and CMFG-cohousing groups. Interestingly, the colonic levels of IL-1β, IL-6, and TNF-α were increased when challenged to DSS only, suggesting that CMFG protect against DSS-induced disturbance of intestinal immune homeostasis. Our findings demonstrated that prophylactic CMFG intervention and microbiota transfer from the CMFG-treated mice decreased the expression of genes related to pro-inflammatory cytokines and oxidative stress. Studies are still needed to explore the role of CMFG in the NF-κB signaling.

## Conclusions

In summary, prophylactic CMFG intervention and microbiota transfer from the CMFG-treated mice prevents DSS-induced gut microbiota dysbiosis, increases fecal SCFAs (propionate and butyrate) and improves the intestinal barrier function. Furthermore, the anti-inflammation effect of CMFG pre-supplementation and transfer of the gut microbiota from CMFG-treated mice was accompanied by improving the stability of gut microbiota and suppressing the accumulation of macrophages in colitis models. Our study provides new insights into the microbiota-mediated regulation of colonic immune cells and provides anti-inflammation therapeutic strategy involving modulation of gut microbiota.

## Materials and methods

### Animals and treatments

Female C57BL/6 mice at 8 weeks of age were obtained from the SPF Biotechnology Co., Ltd, Beijing, China. Throughout the acclimatization and study periods, all animals were maintained on a 12 hr light-dark cycle (21 ± 2°C) under specific pathogen-free conditions with free access to food and water. The functional food ingredients, CMFG, were obtained from Beijing Sanyuan Foods Co. Ltd. Mice in the CMFG + DSS group were orally administered with 100 mg/kg body weight of CMFG solution (MFGM: GOS: FOS = 35.2%: 62.2%: 2.6%; on a daily basis) for 21 days. Mice in the DSS group and Vehicle group underwent daily oral gavage of PBS for 21 days. DSS colitis (DSS group and CMFG + DSS group) was induced by administration of 2.5% DSS (w/v; molecular weight, 36–50 kDa; MP Biomedicals, UK) in drinking water *ad libitum* for 7 days (Figure 1a). Each mouse was scored daily for pathological features, including stool consistency, presence of blood stool, and body weight loss. Individual scores were combined to generate the Disease Activity Index (DAI) which was calculated daily for each mouse. The maximum score was 15 based on assigning a 0–5 scoring system for following parameters.^80^

### Co-housing experiments

Eight-week-old C57BL/6 female mice were daily oral gavage with CMFG solution (100 mg/kg body weight) for 21 days. Meanwhile, the mice were dosed with PBS at the Vehicle group for 21 days. Thereafter, mice in the Vehicle group were continued to receive PBS, while mice in the other groups started to drink 2.5% DSS for 7 days. Meanwhile, mice in the DSS and CMFG + DSS groups were assigned to two subgroups. Half mice in the DSS and CMFG groups were continued to receive PBS (DSS group, *n* = 6) and CMFG (CMFG + DSS group, *n* = 6), respectively. The other half mice were cohoused and continued to receive either PBS (Vehicle-cohousing + DSS group, *n* = 6) or CMFG (CMFG-cohousing + DSS group, *n* = 6) (Figure 6a). During the DSS intervention period, disease activity index (DAI; including stool consistency, presence of blood stool and body weight loss; 0–5 for each parameter) was evaluated daily.^80^

### Tissue collection, fixation and histochemistry

Ileum and colon tissues were fixed in 4% paraformaldehyde, embedded in paraffin, cut into 5-μm-thick sections, and subsequently stained with hematoxylin and eosin (H&E). Images were collected and analyzed using Image J software. Intestinal tissue damage was scored as previously described,^80^ and the epithelial loss of intestinal villi and the infiltration of inflammatory cells were evaluated. The colonic tissues were stained with Alcian Blue (AB) and periodic acid–Schiff (PAS), and the images were collected using microscope (Carl Zeiss AG, Jena, Germany). The acidic mucus-containing goblet cells were counted.

### Transmission electron microscopy (TEM)

The distal colon tissues were washed with 0.9% saline and fixed with glutaraldehyde during the sampling period, and the follow-up preparation steps were performed by the electron microscopy center of China Agricultural University. Images were acquired using a transmission electron microscope (Hitachi Model HT7700, Tokyo, Japan).

### Enzyme-linked immunosorbent assay (ELISA)

Concentrations of IL-1β, IL-6, IFN-γ, TNF-α, myeloperoxidase (MPO), and erythropoietin (EPO) were determined in the plasma and colon samples using ELISA Kits according to the manufacturer’s instructions (Nanjing Jiancheng Biology Engineering Institute, Nanjing, China). The activities of total anti-oxidation capacity (T-AOC), catalase (CAT), the total superoxide dismutase (T-SOD), glutathione peroxidase (GSH-px) and the concentrations of malondialdehyde (MDA) were determined using the commercial kits (Nanjing Jiancheng Biology Engineering Institute, Nanjing, China), and were normalized to total protein levels. These assays were performed according to the manufacturer’s protocol and read at 450 nm using a microplate reader (BioTek Instruments, Inc).

### RNA-seq analysis

Briefly, total RNA was isolated according to the manufacturer’s instructions, and RNA-seq was performed by Majorbio BioTech Co., Shanghai, China. The Illumina HiSeq 2500 platform which was used to construct RNA libraries and generated reads of 125-bp long paired-end (Illumina, San Diego, CA). The read number of each gene was transformed into FPKM (fragment per kilobase of exon model per million mapped reads), and then differentially expressed genes were identified using the DEGseq2 package.^81^ GO enrichment analysis of the DEGs was conducted by the GOseq R package. KEGG pathway enrichment analysis of the DEGs was implemented using the KOBAS software. Raw data files and processed files have been uploaded to the Gene Expression Omnibus public database (GSE161982).

### Flow cytometric analysis

Surgically removed fresh 1-cm mouse colon tissues were opened and washed with cold PBS to remove the fecal contents. The tissues were quickly transported to the centrifuge tube containing 10 mL 1640 medium (10% fetal bovine serum (FBS), 1% penicillin-streptomycin (P/S), 1 mM EDTA) on an orbital shaker at 300 rpm for 30 min at 37°C. After washing, the colons were finely minced and digested with 15 mL of HBSS containing 10% FBS, 1.5 mg/mL Type-VIII Collagenase (C2139; Millipore, Sigma), and 40 μg/mL DNase I at 300 rpm for 15 min at 37°C. After the digestion, the digested colonic lamina propria cells were filtered through a 100-μm strainer, centrifuged at 1500 rpm for 5 min at 4°C, and resuspended in 2 mL PBS for flow cytometric analysis.^82^

The antibodies as follows: (1) CD45-Alexa Fluor 700 (103,128, Biolegend), MHCII-APC (107,613, Biolegend), CD11c-PE (117,307, Biolegend), F4/80-FITC (123,108, Biolegend), CD11b-Percp-Cy5.5 (101,227, Biolegend); (2) CD45-Alexa Fluor 700, CD3-FITC (100,203, Biolegend), CD25-APC (101,909, Biolegend), NK1.1-PE (108,707, Biolegend).

### Immunofluorescence staining

Paraffin embedded 5-μm-thick sections were deparaffinized by heating to 60°C for 15 min, cleared with xylene, followed by an ethanol gradient (75%, 95%, and 100%) and water and steamed for 30 min in citrate buffer for antigen retrieval. BrdU pulse–chase experiments were performed according to the manufacturer’s instructions. In brief, BrdU was intraperitoneally injected (10 μL/g body weight) into mice. After 2 hr, mice were euthanized and incorporated BrdU was detected in the colon tissues. The levels of apoptosis in the colon tissues were detected by TUNEL staining according to the instructions provided as previously described.^83^ The DAPI blue nuclei with the same label were selected as the total cells, and the TUNEL positive cell number per field of intestinal epithelial cells was analyzed. Cell apoptosis was observed by green fluorescence microscopy (200 × magnification).

### 16S rDNA sequencing

Fecal samples for 16S rRNA amplicon sequencing were collected as previously described.^84^ Fecal genomic DNA was extracted from 100 mg frozen fecal samples using the QIAamp® Fast DNA Stool Mini Kit (Qiagen, Hilden, Germany) according to the manufacturer’s protocol. Amplicon libraries covering the V3-V4 distinct regions of the bacterial 16S-rDNA gene were amplified using primers 341 F: 5ʹ-ACTCCTACGGGRSGCAGCAG-3ʹ, and 806 R: 5ʹ-GGACTACVV GGGTATCTAATC-3ʹ. All PCR products were purified using the Qiagen Gel Extraction Kit (Qiagen, Hilden, Germany). Then, the amplicon library was paired-end sequenced (2 × 250) on an Illumina MiSeq platform (Illumina) according to the standard protocols.

Raw fastq files were demultiplexed and quality filtered using QIIME (v.1.17; http://qiime.org/). In brief, the low-quality sequences with a length of <220 nt or >500 nt, an average quality score of <20, and sequences containing >3 nitrogenous bases, were removed. The remaining high-quality sequences were clustered into OTUs at a 97% similarity cutoff using UPARSE (v.7.1; http://drive5.com/uparse/) and chimeric sequences were identified and removed using UCHIME (https://drive5.com/usearch/manual/uchime_algo.html). Taxonomy assignment of OTUs was conducted with the RDP classifier (http://rdp.cme.msu.edu/) against the SILVA 16S rRNA gene database (https://www.arb-silva.de/) using confidence threshold of 70%.

### SCFAs quantitative analysis

The concentrations of fecal SCFAs (acetate, propionate, and butyrate) were detected with an Ion chromatographic method as previously described.^85,86^ Briefly, 10 mg of fecal sample was added in a screw-capped tube with 160 μL of distilled water (Thermo Scientific, Waltham, MA). The mixture was treated with a 30 min ultrasonic bath and then centrifuged at 8,000 g for 10 min at 4°C. One milliliter of supernatant was diluted with water (1:50), and filtered with 0.22 μm mesh. The extracted samples were kept in a 2 mL screw-cap vial and finally injected into a Dionex ICS-3000 Ion Chromatography System (Dionex, Sunnyvale, CA, USA).

### RNA extraction and real-time PCR

The colon tissues were immediately snap-frozen in liquid nitrogen and homogenized. Total RNA was isolated from the tissues using RNeasy Mini Kit (QIAGEN) according to the manufacturer’s instructions. The expression was analyzed in triplicate on one plate per gene. The mRNA abundance of target genes was normalized using an internal control (β-Actin) and calculated using the 2^−ΔΔCt^ method. All primers were synthesized by Invitrogen Life Technologies (Invitrogen, Shanghai, China), which are shown in Supplementary Table S1.

### Statistical methods

All data were represented as means ± SEM. Statistical analysis, excluding microbiome, was performed using Prism 8.0 (GraphPad Software, San Diego, CA). Data from more than two groups were compared using one-way ANOVA followed by Tukey’s multiple comparison tests. *P* ≤ 0.05 was considered statistically significant.

The alpha diversity (Shannon’s diversity index and Sob’s diversity index) was determined by sampling-based operational taxonomic unit (OTU) analysis and presented by observed OTU, which was calculated using the MOTHUR program (version v.1.30.1). Principal coordinates analysis (PCoA) plots were generated on the basis of Bray-Curtis dissimilarity using the R (https://www.R-project.org/) package phyloseq, and clustering analysis was performed by PERMANOVA using the R package vegan. The predominance of bacterial communities between groups were analyzed by linear discriminant analysis (LDA) effect size method. At the species level, relative abundance for organism was calculated as follows: relative abundance = (number of unique alignment positions in genome × 1,000,000)/(number of total aligned bacterial reads × genome size). The relative abundance values were then per-sample normalized such that the total relative abundance for each sample summed to one.^87,88^ Based on the normalized relative abundance matrix, features with significantly different abundances between assigned taxa were determined by linear discriminant analysis effect size (LEfSe) with the Kruskal-Wallis rank-sum test (*p* < .05) and LDA was used to assess the effect size of each feature.

## Supplementary Material

Supplemental Material

## Data Availability

The bacterial 16S rDNA sequences were deposited to the sequence read archive. The Bioproject accession for the 16S sequencing data reported in this paper is PRJNA679681.

## Abbreviations

IBD: Inflammatory bowel disease; DSS: Dextran sodium sulfate; MFGM: Milk fat globule membrane; FOS: Fructo-oligosaccharides; GOS: Galacto-oligosaccharides; CMFG, Containing MFGM, FOS and GOS; BW: Body weight; SCFAs: Short-chain fatty acids; DAI: Disease activity index; UC: Ulcerative colitis; CD: Crohn’s disease; HMOs: Human milk oligosaccharides; FMT: Fecal microbial transplantation; AB: Alcian Blue; PAS: Periodic acid–Schiff; HE: Hematoxylin and eosin; TEM: Transmission electron microscopy; ELISA: Enzyme-linked immunosorbent assay; MPO: Myeloperoxidase; EPO: Erythropoietin; T-AOC: Total anti-oxidation capacity; CAT: Catalase; T-SOD: Total superoxide dismutase; GSH-Px: Glutathione peroxidase; MDA: Malondialdehyde; GO: Gene Ontology; KEGG: Kyoto Encyclopedia of Genes and Genomes; FBS: Fetal bovine serum; BrdU: Bromodeoxyuridine; TUNEL: terminal deoxynucleotidyl transferase-mediated dUTP nick-end labeling; OTU: Operational taxonomic unit; PCoA: Principal coordinates analysis; LDA: Linear discriminant analysis; LEfSe: Linear discriminant analysis effect size. MHC: major histocompatibility complex; TNF: tumor necrosis factor; TH1: type 1 T helper; Treg: regulatory T; iNOS: inducible nitric oxide synthase; ROS: reactive oxygen species.
