# Supplementary material for: Cohousing-mediated microbiota transfer from milk bioactive components-dosed mice ameliorate colitis by remodeling colonic mucus barrier and lamina propria macrophages
Source: Gut Microbes. 2021 Mar 31;13(1):1903826. doi: 10.1080/19490976.2021.1903826 (PMC8018355; doi:10.1080/19490976.2021.1903826)
Supplement: Supplemental Material [file KGMI_A_1903826_SM8819.zip › Supplementary information/Supplementary legends.docx]

**Figure S1.** Prophylactic CMFG intervention improved inflammatory and oxidation profile in the colon. (A-D) The levels of the inflammation-related index in the colon were analysed by ELISA Kits. (E-I) The levels of the oxidative-related index in the colon were analysed. Asterisks denote significant differences (**p* ≤ 0.05, ** *p* ≤ 0.01, *** *p* ≤ 0.001), *n* = 6 per group, data are represented as mean ± SEM.

**Figure S2.** Prophylactic CMFG intervention improved inflammation, oxidative, and barrier function genes expression in the colon. (A-E) The mRNA expression levels of the inflammation-related genes in the colon were analysed by RT-qPCR. (F-H) The mRNA expression levels of the oxidative-related genes in the colon were analysed. (I-M) The mRNA expression levels of the intestinal barrier function-related genes in the colon were analysed. Asterisks denote significant differences (**p* ≤ 0.05, ** *p* ≤ 0.01, *** *p* ≤ 0.001), *n* = 6 per group, data are represented as mean ± SEM.

**Figure S3.** Cohousing of CMFG-dosed mice improved inflammatory and oxidation profile in the colon. (A-D) The levels of the inflammation-related index in the colon were analysed by ELISA Kits. (E-I) The levels of the oxidative-related index in the colon were analysed. Asterisks denote significant differences (**p* ≤ 0.05, ** *p* ≤ 0.01, *** *p* ≤ 0.001), *n* = 6 per group, data are represented as mean ± SEM.

**Figure S4.** Gut microbiota structure of CON mice, DSS mice, and CMFG + DSS mice at different points in time (at day 0, at day 3 and at day 7).

**Figure S5.** Gut microbiota structure of DSS mice, CMFG + DSS mice, Vehicle-cohousing mice and CMFG-cohousing + DSS mice (at day 3 and at day 7).
