# Supplementary material for: Cohousing-mediated microbiota transfer from milk bioactive components-dosed mice ameliorate colitis by remodeling colonic mucus barrier and lamina propria macrophages
Source: Gut Microbes. 2021 Mar 31;13(1):1903826. doi: 10.1080/19490976.2021.1903826 (PMC8018355; doi:10.1080/19490976.2021.1903826)
Supplement: Supplemental Material [file KGMI_A_1903826_SM8819.zip › Supplementary information/Supplementary Table S1.docx]

Supplementary Table S1. Mice primer sequences in RT- PCR assays.

| **Genes** | **Forward** | **Reverse** |
| --- | --- | --- |
| mβ-Actin | TGGAATCCTGTGGCATCCATGAAAC | TAAAACGCAGCTCAGTAACAGTCCG |
| mTNF-α | TGGGAGTAGACAAGGTACAACCC | CATCTTCTCAAAATTCGAGTGACAA |
| Mifn-γ | GCGGCTGACTGAACTCAGATTGTAG | AGTGCTGTCTGGCCTGCTGTTA |
| mIL-1β | CTCGCAGCAGCACATCAACAAG | GGAAGGTCCACGGGAAAGACAC |
| mIL-6 | ACCACGGCCTTCCCTACTT | CACAACTCTTTTCTCATTTCCAC |
| mIL-10 | CCCTTTGCTATGGTGTCCTT | TGGTTTCTCTTCCCAAGACC |
| miNOS | CATCACCACGCCTCCAACTCAG | AGTCTCAAGCCTCTGCCTCTCG |
| mNF-κB | GACACGACAGAATCCTCAGCATCC | CCACCAGCAGCAGCAGACATG |
| mTLR4 | GGTGTGAAATTGAGACAATTGAAAAC | GTTTCCTGTCAGTACCAAGGTTGA |
| mZO-1 | CGGAACTATGACCATCGCCTAC | CTTCGGGATGTTGTCTGGAGTC |
| mClaudin-1 | AGCTGTGCATGGCCTCTTGT | CCAATGTCAATGGCAACACCCT |
| mOccludin | CAGCCTCGGTACAGCAGCAAT | ATAGTGGTCAGGGTCCGTCCTC |
| mMUC1 | AATGGCTCCTCGGTGCTACCTA | TGACTTGGCACTGAAGGCTGAG |
| mMUC2 | TGCTGACGAGTGGTTGGTGAATG | GATGAGGTGGCAGACAGGAGACA |
