# Supplementary figures and images for: Cohousing-mediated microbiota transfer from milk bioactive components-dosed mice ameliorate colitis by remodeling colonic mucus barrier and lamina propria macrophages
Source: Gut Microbes. 2021 Mar 31;13(1):1903826. doi: 10.1080/19490976.2021.1903826 (PMC8018355; doi:10.1080/19490976.2021.1903826)

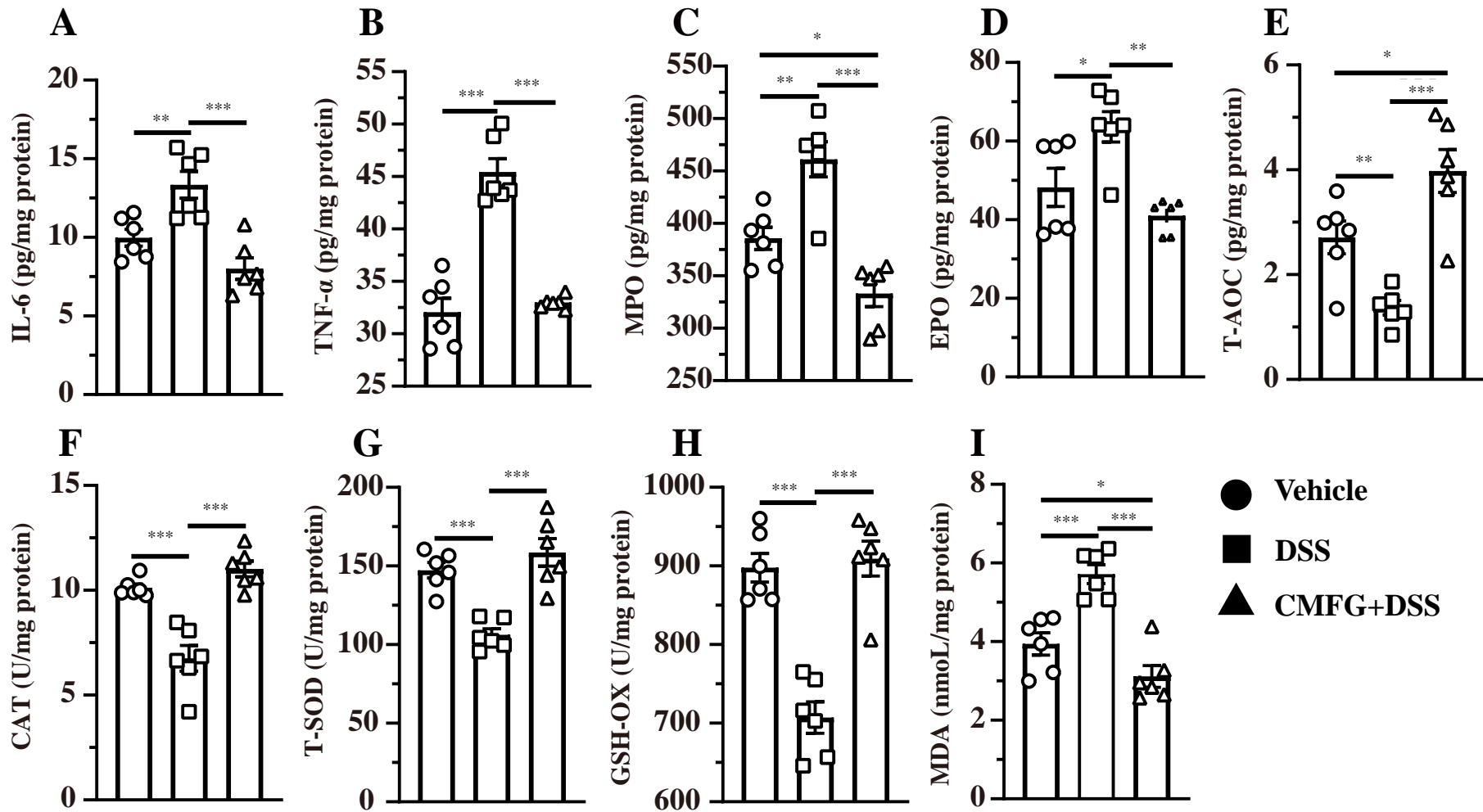

Supplement: Supplemental Material [file KGMI_A_1903826_SM8819.zip › Supplementary information/Figure S1.pdf]

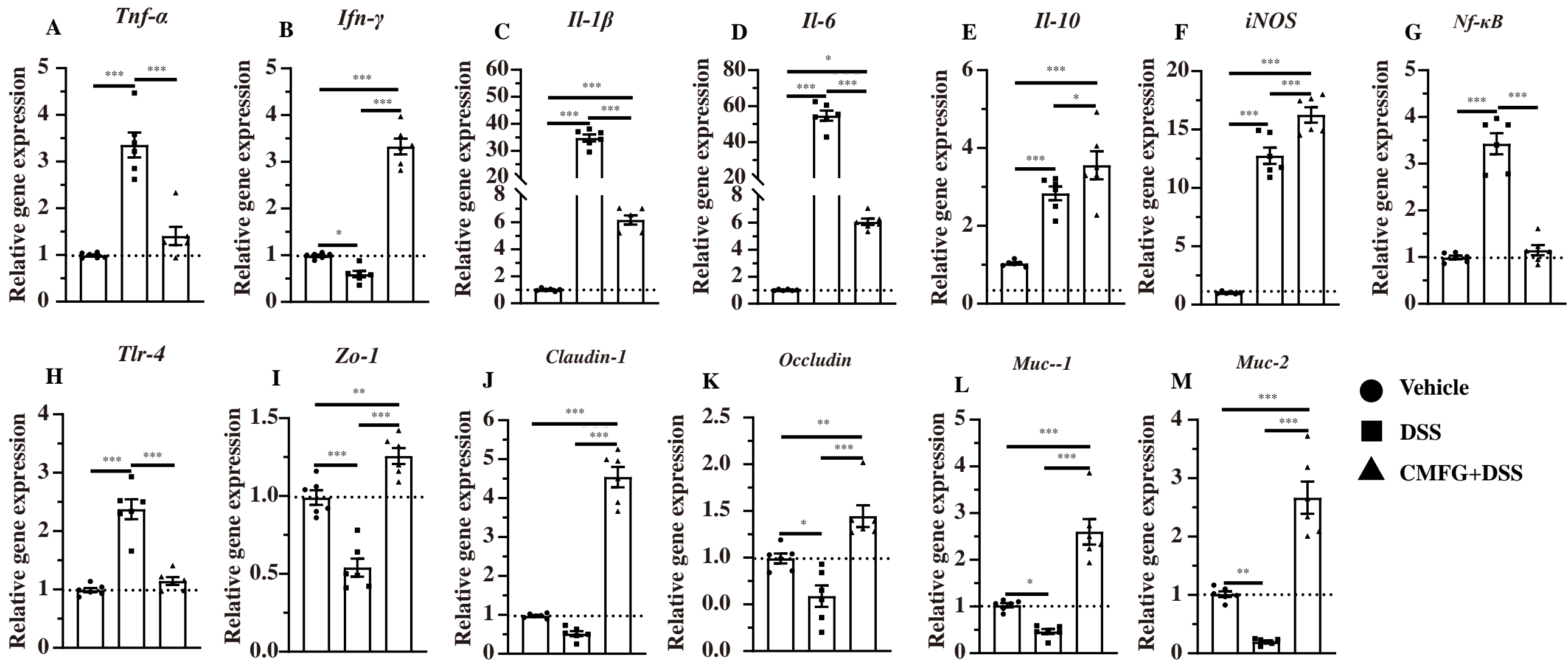

Supplement: Supplemental Material [file KGMI_A_1903826_SM8819.zip › Supplementary information/Figure S2.pdf]

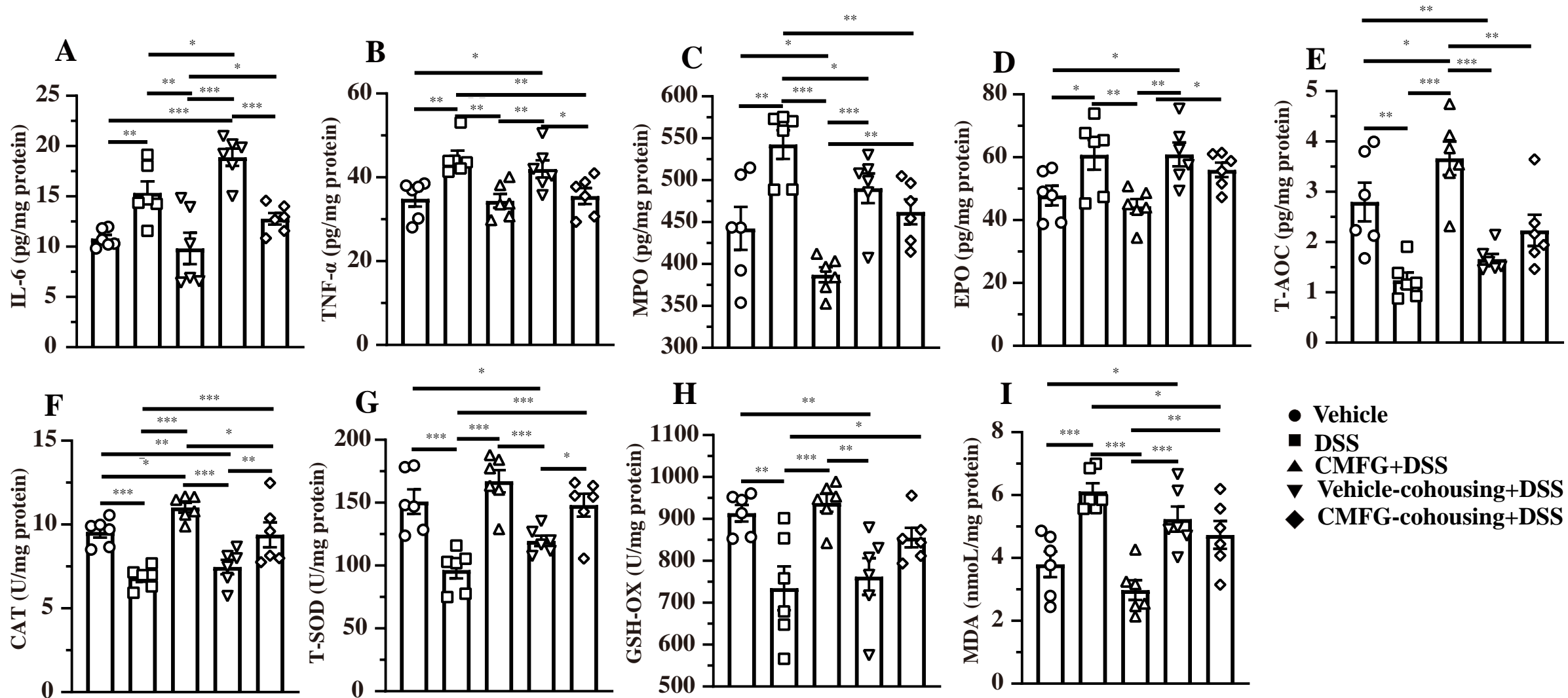

Supplement: Supplemental Material [file KGMI_A_1903826_SM8819.zip › Supplementary information/Figure S3.pdf]

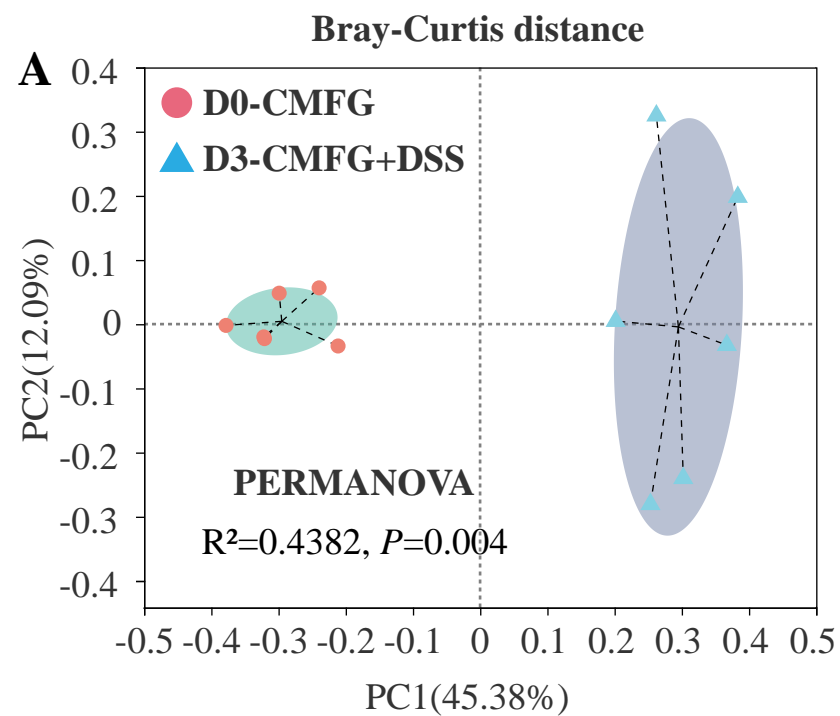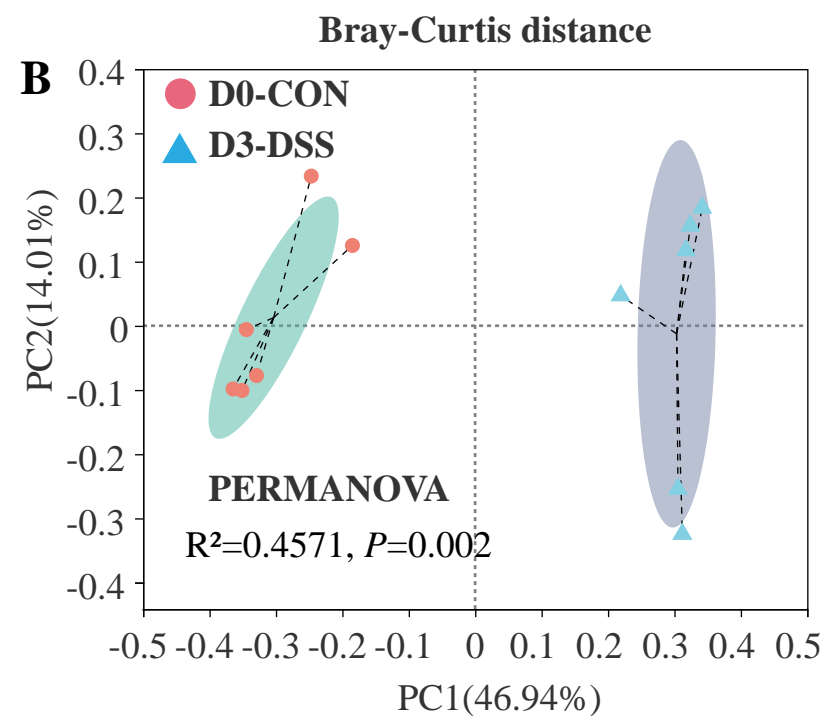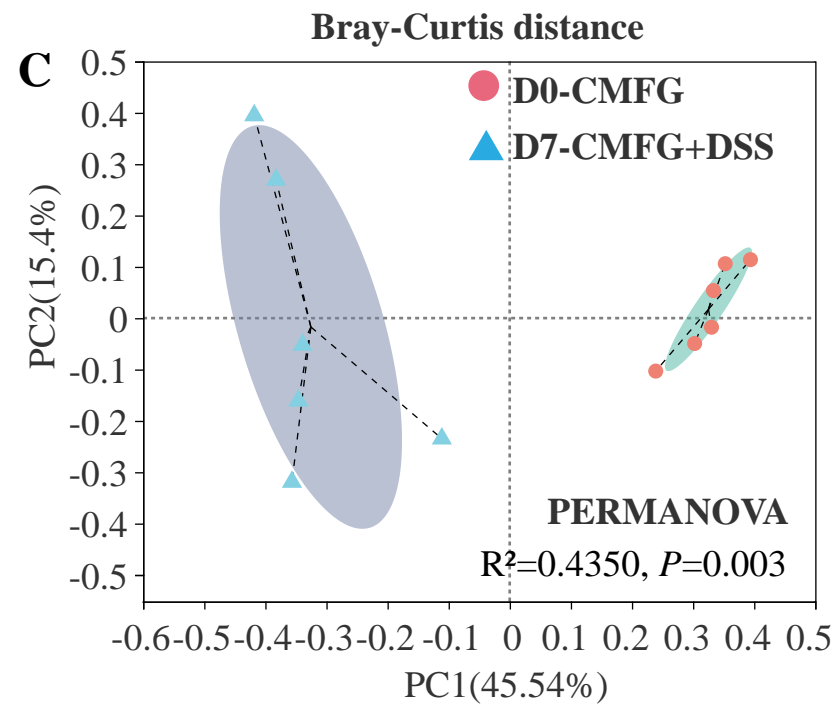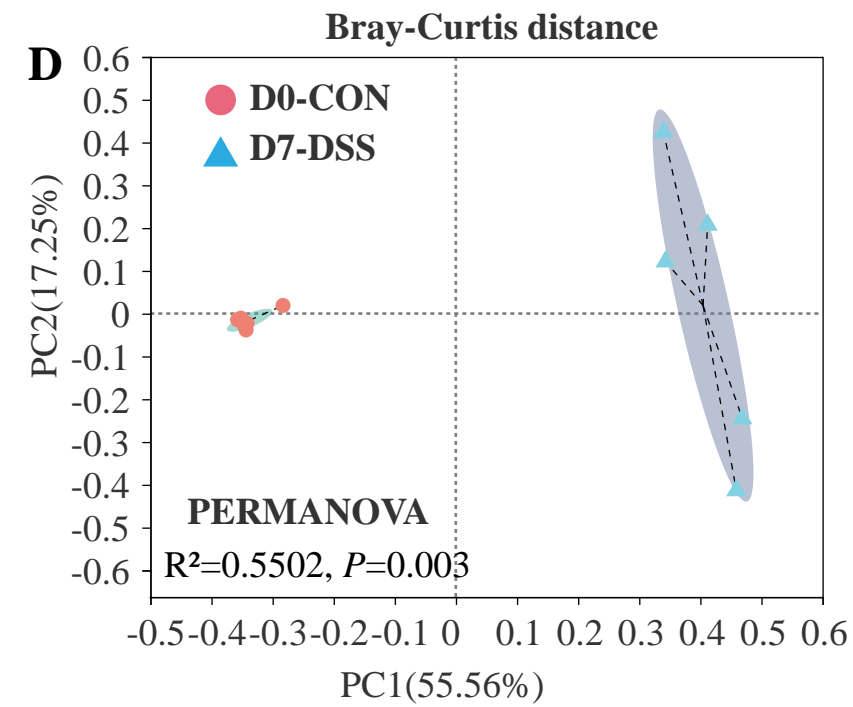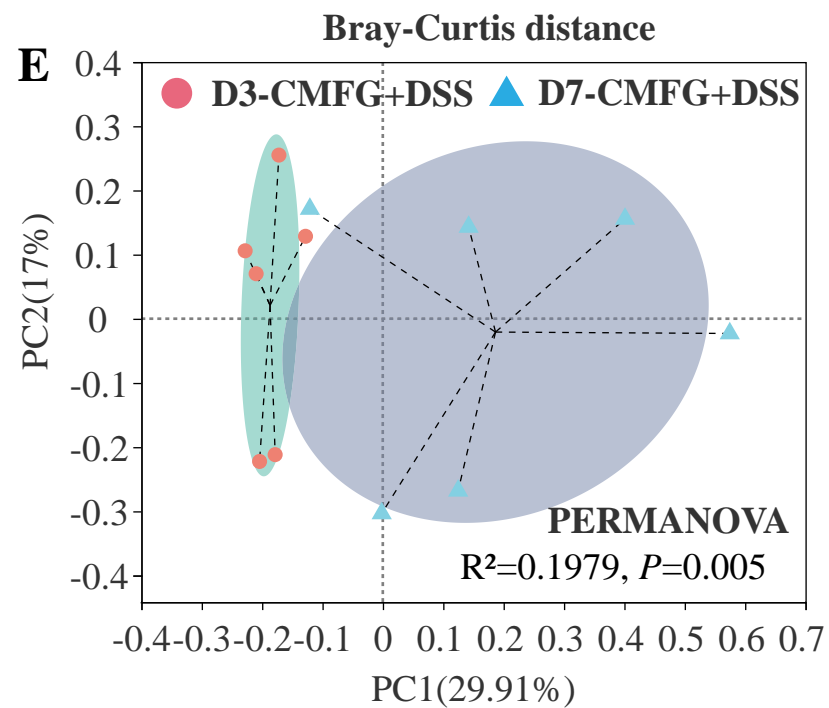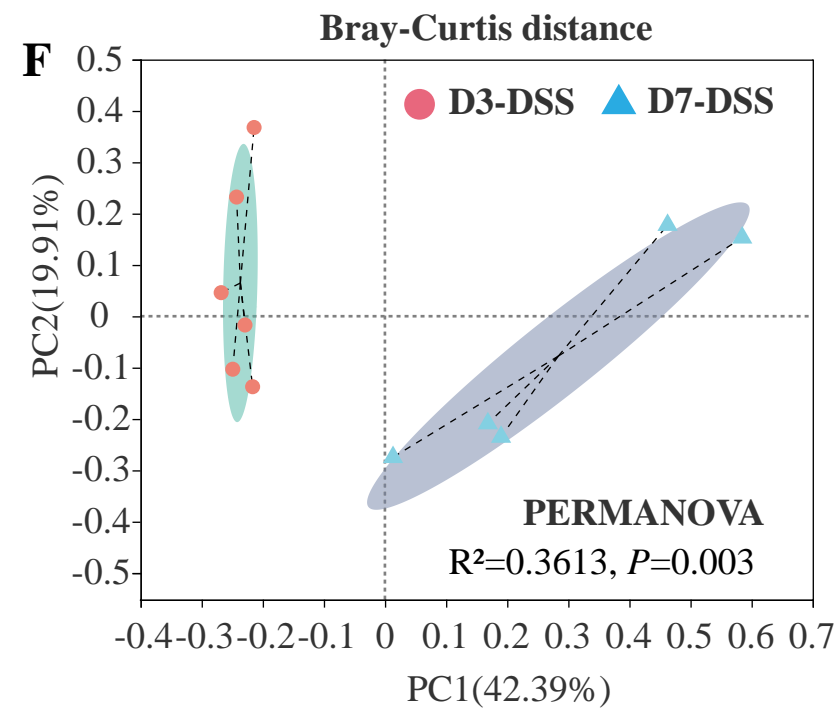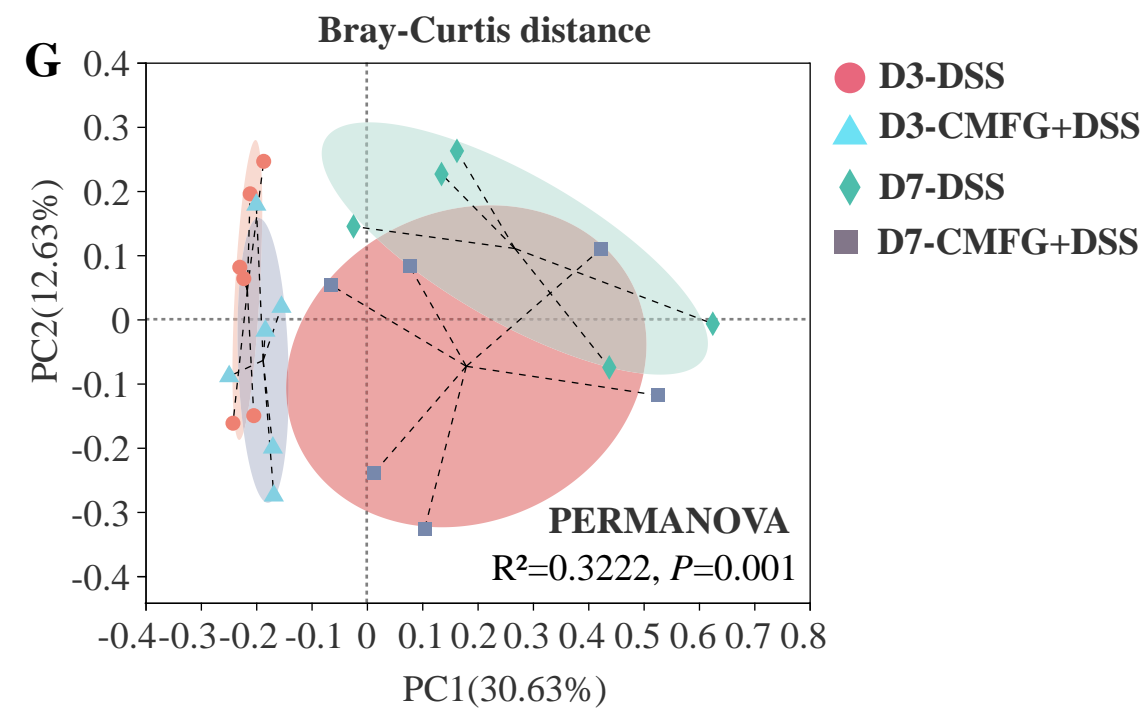

Supplement: Supplemental Material [file KGMI_A_1903826_SM8819.zip › Supplementary information/Figure S4.pdf]

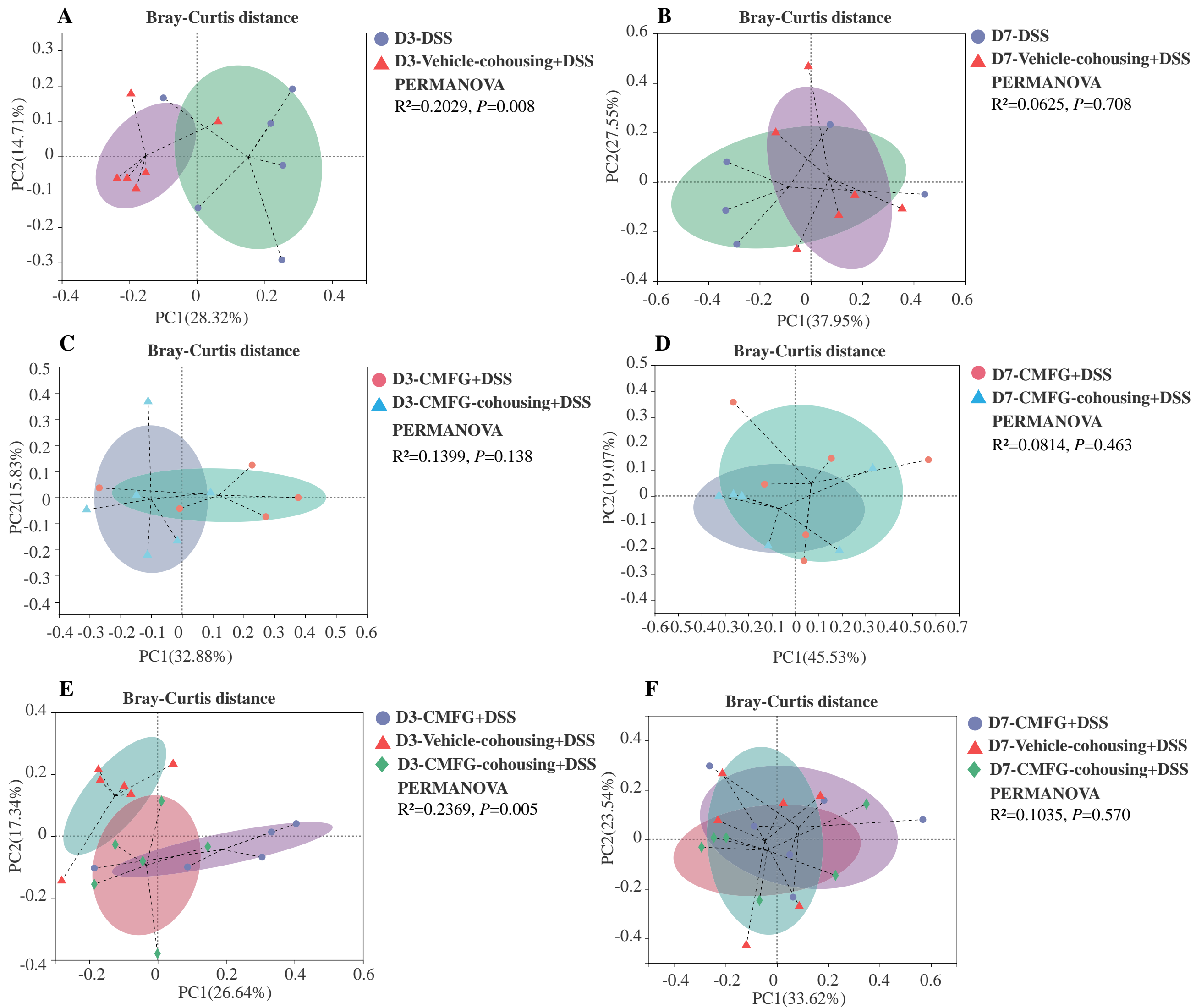

Supplement: Supplemental Material [file KGMI_A_1903826_SM8819.zip › Supplementary information/Figure S5.pdf]
